# Assessing the universal health coverage target in the Sustainable Development Goals from a human rights perspective

**DOI:** 10.1186/s12914-016-0106-y

**Published:** 2016-12-15

**Authors:** Audrey R. Chapman

**Affiliations:** University of Connecticut School of Medicine, 263 Farmington Ave, Farmington, CT 06030 USA

**Keywords:** Universal health coverage, Rights-based, Financial risk protection, Access to health services, Priority to the disadvantaged

## Abstract

**Background:**

The UN’s Sustainable Development Goals (SDGs), adopted in September 2015, include a comprehensive health goal, “to ensure healthy lives and promote well-being at all ages.” The health goal (SDG 3) has nine substantive targets and four additional targets which are identified as a means of implementation. One of these commitments, to achieve universal health coverage (UHC), has been acknowledged as central to the achievement of all of the other health targets. As defined in the SDGs, UHC includes financial risk protection, access to quality essential health-care services, and access to safe, effective, quality and affordable essential medicines and vaccines for all.

**Discussion:**

This article evaluates the extent to which the UHC target in the SDGs conforms with the requirements of the right to health enumerated in the International Covenant on Economic, Social and Cultural Rights, the Convention on the Rights of the Child, and other international human rights instruments and interpreted by international human rights bodies. It does so as a means to identify strengths and weaknesses in the framing of the UHC target that are likely to affect its implementation.

**Summary:**

While UHC as defined in the SDGs overlaps with human rights standards, there are important human rights omissions that will likely weaken the implementation and reduce the potential benefits of the UHC target. The most important of these is the failure to confer priority to providing access to health services to poor and disadvantaged communities in the process of expanding health coverage and in determining which health services to provide. Unless the furthest behind are given priority and strategies adopted to secure their participation in the development of national health plans, the SDGs, like the MDGs, are likely to leave the most disadvantaged and vulnerable communities behind.

## Background

There has been a growing commitment in recent years to the goal of achieving universal health coverage (UHC). UHC has been identified as potentially the third global health transition, the first being public health improvements such as basic sewage and sanitation and the second, the epidemiological transition that reduced the toll of communicable diseases [1]. Major health and development organizations, including the World Health Organization, the World Bank Group, the Rockefeller Foundation, Oxfam, the Gates Foundation, the International Labour Organization, and the United Nations Children’s Fund (UNICEF), have endorsed initiatives promoting UHC [2]. Dr. Margaret Chan, the World Health Organization (WHO) Director General, has described universal health coverage as the single most powerful concept that public health has to offer [3].

Thus it is not surprising that UHC was selected as one of the health targets in the United Nations Sustainable Development Goals (SDGs) adopted in September 2015 to implement the inclusive health goal (Goal 3), “to ensure healthy lives and promote well-being at all ages” [4]. Target 3.8 is to “achieve universal health coverage, including financial risk protection, access to quality essential health-care services and access to safe, effective, quality and affordable essential medicines and vaccines for all” [5]. Although universal coverage is only one of nine substantive targets and four additional targets labeled as means of implementation that are related to Goal 3, it is considered to be the target that underpins and is key to the achievement of all the others [6]. UHC also receives special attention in the Declaration for Transforming Our World endorsed by heads of government that precedes the identification of the SDGs: UHC is linked with the central commitment in the SDGs to leave no one behind: “To promote physical and mental health and well-being, and to extend life expectancy for all, we must achieve universal health coverage and access to quality health care. No one must be left behind” [7].

Although a human rights approach also has a commitment to UHC, various paths to UHC and the way the goal is conceptualized are not necessarily consistent with international human rights principles. This article evaluates the extent to which the UHC target in the SDGs conforms with the requirements of the right to health as enumerated in the International Covenant on Economic, Social and Cultural Rights [8] (ICESCR) and the Convention on the Rights of the Child [9] and interpreted in key documents, particularly the United Nations Committee on Economic, Social and Cultural Rights in its 2000 general comment interpreting the right to health [10]. It does so as a means to identify strengths and weaknesses in the framing of the UHC target that are likely to affect its implementation.

The human rights community generated some of the most sustained criticism of the manner in which the Millennium Development Goals (MDGs), the predecessor set of international development goals to the SDGs, were designed, framed, and monitored. Human rights critiques went beyond the failure of the MDGs to explicitly incorporate human rights norms and commitments. One concern was that the globally fixed targets in the MDGs would allow middle-income countries to evade major responsibility for implementation. Another was that by failing to require disaggregation in monitoring and reporting the MDGs encouraged governments to focus solely or primarily on raising national percentages. In the process, states were tempted to cherry-pick implementation by focusing on more advantaged groups which were easier and cheaper to reach to the exclusion of minorities, persons with disabilities, or the poorest of the poor. Others believed that the MDGs focused on achieving quantified targets at the expense of quality. Another strand of criticism cited the inadequacy of the indicators selected to monitor the MDGS which then became used as planning tools at the expense of human rights commitments [11]. These criticisms and concerns anticipated deficiencies that affected the implementation of the MDGs. The analysis in this article suggests that the SDGs have many of the same problems.

There are various rhetorical commitments to human rights in the SDGs. The Declaration preceding Transforming our world: the 2030 Agenda for Sustainable Development envisions a world of universal respect for human rights and human dignity [12]. It also states that the Agenda is grounded in the Universal Declaration of Human Rights and international human rights treaties [13]. In addition, the Declaration “reaffirms the importance of the Universal Declaration of Human Rights, as well as other international instruments relating to human rights and international law” and “the responsibility of all states in conformity with the Charter of the United Nations, to respect, protect and promote human rights without distinction as to race, colour, sex, language, religion, political or other opinion, national or social origin, property, birth, disability or other status” [14].

Of the various targets related to Goal 3, the universal health coverage target arguably reflects the right to health the most closely. UHC has been termed “a practical expression of the right to health” [15]. It is explicitly enumerated as a core obligation related to children’s right to health [16], and the commitment to universality in access to key health services is implicit in other international and regional human rights instruments. Indeed, some health and human rights advocates had earlier proposed replacing the various health-related goals in the MDGs with a single overarching health goal of UHC in the SDGs, provided that the goal would include a straightforward confirmation that international assistance is essential, not optional [17]. Significant progress toward UHC, consistent with the requirements of the right to health, could have the potential of enabling the approximately one billion people currently estimated to not have access to the health services they need the opportunity to obtain them and to do so affordably.

Nevertheless, the 2030 Agenda for Sustainable Development is not and does not purport to be a human rights document. Despite the commitment to the principle “no one left behind,” none of the SDG goals or targets, including target 3.8, is framed as a human rights entitlement. While the four components of target 3.8 overlap with dimensions of the right to health, as interpreted in UN Committee on Economic, Social and Cultural Rights’ General Comment No. 14 (GC 14) [18], there are important human rights omissions as well. The failure to incorporate the human rights principles noted below is likely to weaken and perhaps undermine the achievement of UHC.

There were efforts to promote a human rights orientation to the SDG health goal, most notably by the Go4HealthProject, a consortium of academics and civil society members tasked with advising the European Commission on the international health-related goals to follow the MDGS. In addition, WHO published a policy brief “Anchoring universal health coverage in the right to health: What difference would it make?” The paper states that for WHO UHC is a practical expression of the concern for health equity and the right to health and thus advances the overall objective of the WHO, the attainment by all peoples of the highest possible standard of health as a fundamental right. Without specific reference to the SDGs, which may not have as yet been adopted, but presumably with the SDGs in mind, the paper argues that efforts toward achieving UHC promote some, but not all, of the efforts required to achieve the right to health [19].

A Go4Health study based on interviews in 2013 and 2014 with participants from key multilateral and other organizations which played an important role in the framing of the post-2015 health goals identified several reasons why the right to health and for that matter other human rights failed to to be incorporated in the SDGs. Some respondents expressed concern that attempting to integrate human rights into the post-2015 document would result in decision-making delays. There was unease around sexual and reproductive health rights as a ‘fault line’ and especially to its connection to debates around the rights of lesbian, gay, bisexual, and transsexual communities. An overarching post-2015 right to health goal was seen to be too broad to be defined despite acknowledgement by at least some that the right to health was well-articulated in international law. Even if a right to health goal was incorporated, it was considered too difficult to operationalize and practically to implement [20].

## Discussion

### Overlap of UHC with the right to health

As noted, under some circumstances the goal of universal health coverage can be considered to be an expression of the right to health. The preamble in a 2011 World Health Assembly resolution calling for the adoption of UHC by member countries specifically links the achievement of UHC to article 25.1 of the Universal Declaration of Human Rights [21], which enumerates the right to a standard of living adequate for health and well-being including medical care. So does a 2012 UN General Assembly resolution which called on states to realize UHC while reaffirming the right to health [22].

#### Universal access

But universal access, which is a right to health requirement, is a concept that includes but goes beyond universal coverage. Universal access implies the absence of any geographic, financial, organizational, sociocultural, and gender-based barriers to care [23]. The first of the core rights to health obligations listed in General Comment 14, which is incumbent on all state parties (the 163 states which have ratified the International Covenant on Economic, Social and Cultural Rights) to achieve immediately, is “to ensure the right of access to health facilities, goods and services on a non-discriminatory basis, especially for vulnerable or marginalized groups” [24]. Simply expanding health coverage, especially if it continues to exclude poor and vulnerable communities, is not sufficient from a human rights perspective.

#### Financial risk protection

Financial risk protection also overlaps with right to health requirements. According to General Comment 14, economic accessibility, conceptualized as health facilities, goods and services being affordable for all whether privately or publicly provided, including for socially disadvantaged groups, is an essential element of the right to health [18]. Again, the human rights standard proposed is more stringent than the requirements of target 3.8. The text of the general comment further notes that equity considerations demand that poorer households should not be disproportionately burdened with health expenses as compared to more affluent households [18]. This implies either that health services, at least basic health services, will be provided free of cost or that poor and disadvantaged groups will be heavily subsidized. The SDGs do not have this requirement.

#### Access to quality health services and affordable essential medicines

The second dimension of target 3.8, access to quality essential health services and access to safe, effective, quality and affordable essential medicines and vaccines, also has a human rights counterpart. The creation of conditions which would assure to all medical service and medical attention in the event of sickness is one of the four steps the International Covenant on Economic, Social and Cultural Rights enumerates for state parties to undertake to realize the right to health [25]. Similarly, the Convention on the Rights of the Child has a provision to ensure necessary health care to all children with an emphasis on the development of primary care and another provision to ensure appropriate prenatal and postnatal health care for expectant mothers [26]. These requirements are framed as legal obligations for all state parties, and not as optional goals, albeit with a recognition that they will often need to be implemented gradually with steps taken to the maximum of available resources [27].

General Comment 14 also enumerates the provision of essential drugs, as defined in the WHO Action Programme on Essential Drugs, as a core obligation for state parties [28]. Anand Grover, the second Special Rapporteur for the right to health, specified in his 2009 report that states have an obligation under the right to health to ensure that medicines are available, financially affordable, and physically accessible to everyone in their jurisdiction [29]. Currently, approximately two billion people, some one-third of the world’s population, do not have access to the medicines necessary for their health care [30].

#### Monitoring

Both human rights and the SDGs acknowledge the importance of monitoring to evaluate progress toward implementation of goals. However, there is a major difference in their methodologies. A human rights approach requires systematic monitoring initiatives using disaggregated data in order to identify which groups and communities are benefitting and which are being left behind,. Country level percentages do not suffice for this purpose. WHO’s proposed monitoring framework for UHC also sought to produce statistics to highlight health inequalities by major stratifiers, including demographic (age, sex/gender), socioeconomic status (wealth, education), geography (province/district) and other characteristics (migration, minorities etc.) [31]. While the February 2016 report of the UN’s Inter-Agency and Expert Group on Sustainable Development Indicators, composed of representatives of national statistical offices, acknowledged the need for data disaggregation for effective monitoring [32], it did not incorporate any recommended disaggregation for the indicators for UHC [33]. Instead its approach was to monitor the average country level coverage of a list of health services and to enumerate the number of people covered by health insurance or a public health system per 1000 population [34]. Unless the indicators and monitoring strategy change, the SDGs will replicate many of the monitoring shortcoming of the MDGs.

### Absence of key human rights principles and safeguards

Importantly, there are many components of a human rights approach to universal health coverage that are absent in target 3.8 and the SDGs more broadly. Despite the commitment to the principle “no one left behind,” none of the SDG goals or targets, including target 3.8, is framed as a human rights entitlement applicable to all members of the population and enabling those left behind to seek a remedy. Nor are the human rights principles of human dignity, equality, and nondiscrimination incorporated in the SDGs. While human rights are framed as legal obligations applicable to the countries that have ratified the relevant instruments or incorporated the entitlement into law, there is nothing compulsory about implementing the SDGs. The SDG Declaration characterizes its goals as global in nature and universally applicable but notes that each government will set its own targets taking national circumstances into account [35]. Contrary to the human rights norm of participation, there is no requirement in the SDGs that governments consult their population in determining priorities for expanding coverage or framing policies for implementation. Nor is there recourse for individuals or populations for the failure of governments to implement goals and targets. A human rights approach would have provided safeguards to truly ensure that no one gets left behind.

#### Absence of priority to the disadvantaged

Human rights are predicated on giving priority to the poor and disadvantaged. In human rights documents the groups identified as deserving of special attention and protection include the economically poor and other groups who have previously been excluded, overlooked, and discriminated against such as women, children, the elderly, disabled people, ethnic and racial minorities, and indigenous peoples. The need to undertake special measures in health-related policies, planning, programmes, and research in order to promote the right to health of these groups is highlighted in the UN Committee on Economic, Social and Cultural Rights’ general comment interpreting the right to the highest attainable standard of health [36]. One of the core obligations incumbent on all state parties is to ensure the right of access to health facilities, goods and services, especially for vulnerable or marginalized groups [37]. Applying this norm to human rights and ethical requirements for expanding coverage on the path to UHC, a report of the WHO Consultative Group on Equity and Universal Health Coverage emphasizes the importance of the principle of fair distribution, specifically that coverage and use of health services should be based on need and priority should be given to policies benefiting the worse-off groups. In particular, according to the report, no one should be denied access to high-priority services because he or she is too poor to be able to pay for them [38].

There are no such commitments in the SDGs. This is problematic. As Darius Puras, the current Special Rapporteur on the right to health, has noted the introduction of UHC without targeted measures to confer priority to the poor and marginalized in the expansion of coverage and in developing priorities as to which services to provide risks entrenching inequality. In countries lacking strong health systems governments may pursue strategies disproportionately benefiting privileged groups [39]. For example, a UHC progress analysis of 11 countries at different levels of development shows that UHC expansion usually begins with civil servants or urban formal sector workers, and poorer people often initially lose out. Further skewing the benefits of UHC, the clinical sector commonly favors expensive specialized health services primarily accessible to a small, privileged fraction of the population [40]. In contrast, the right to health recognizes the importance of prioritizing investments in primary and preventive care so as to reach a far larger sector of the population [41].

Even though the SDGs have a goal to reduce inequalities within and between countries (Goal 10), this principle does not have a health component. Nor is it applied to the implementation of the SDGs. Without an explicit commitment to confer priority on poor and disadvantaged individuals and communities and in the absence of a requirement to monitor targets on a disaggregated basis, low and middle-income countries seeking to improve their health coverage rates are likely to do so in the easiest and least expensive manner by focusing efforts in their more developed areas where there is already health infrastructure in place. This is what precisely happened during the MDG process. According to the 2015 UN MDG Report, despite the many successes in implementing the MDGS, the poorest and most disadvantaged people were being left behind. The report also observed that targeted efforts would be needed to reach these groups [42]. If the effort to expand coverage in a specific country were to display a trickle-down pattern of spread marked by increases first in better-off areas and groups, as has been the case in many countries to date, people who are poor and predominantly located in rural and less accessible areas could gain little from the UHC target.

As a recent Overseas Development Institute report points out, governments need to confer priority to efforts to improve the lives of the poorest and most marginalized people from the beginning of initiatives to implement the SDGs if these groups are not to be left behind, preferably in the first 1000 days or 3 years of the SDG process. The longer governments wait to implement pro-poor and pro-marginalised policies, the harder it will be for them to deliver on the SDG goals by 2030, the SDG deadline. This is because the amount of effort needed to compensate for every 3 years of inaction will increase exponentially. For example, if countries in Africa, which currently need to reduce preventable child deaths at a rate of 7% a year between 2015 and 2030 to meet the SDG target, wait until 2018 before taking action the rate will increase to 9% a year, and delaying still further until 2027 will require them to reduce child mortality more than four times faster than if they were to start today – which would be an impossible task [43].

To assure that vulnerable groups are fully included within and benefit from the progressive realization of UHC also requires the careful design and implementation of the basic architecture of the health system and the adoption of strategies for advancing toward universal coverage that target improving disadvantaged communities access to health care. After decades of inadequate funding and insufficient investment in health institutions and services the health systems of many countries are seriously weakened and sometimes dysfunctional. Often these problems disproportionately affect poor and disadvantaged communities. According to WHO, the health systems in many countries remain underfunded and struggle to provide even basic health services. Access to health services is particularly poor for rural areas and the poorest populations. Also many facilities deliver substandard care. In addition, many countries continue to face major shortages of trained health workers, particularly in rural areas [44]. If countries are to transition to UHC on a meaningful and inclusive basis, there is an urgent need for many to invest in health system strengthening particularly in rural areas and less developed regions, but there is no such target in the SDGs.

#### Financial protection

The way in which financial risk protection is conceptualized and the policies adopted to implement this objective also have important repercussions for poor and disadvantaged groups. The SDGs do not provide guidance on this matter. Two priorities of pro-poor financial risk protection are to significantly reduce or preferably eliminate out-of-pocket fees for health services, at least for primary health care services, and to provide protection from catastrophic expenditures. Out-of-pocket user fees, which are a dominant source of financing for health care in low-income countries, have a disproportionate impact on the poor who must pay considerably larger proportions of their incomes for health care than more affluent households. Every year some 100 million people, most of whom live in low-income countries, are pushed into poverty as a result of excessive or catastrophic spending on health care [45]. Therefore providing protection from catastrophic health expenditures, calculated as the proportion of people who spend more than 40% of their incomes on health related costs, is a high priority.

It is troubling that the indicators selected at the time of writing by the UN’s Inter-Agency and Expert Group on Sustainable Development Indicators are not adequate to assess the impoverishing effect of health spending on the poorest and most marginalized groups. The indicators selected for monitoring played an important role in determining policy priorities in the MDG process and they are likely to do so for the SDGs as well. At the February 2016 meeting of the Inter-Agency and Expert Group on Sustainable Development Indicators the proposed indicator was changed from the fraction of the population protected against catastrophic/impoverishing out-of-pocket health expenditure [46] to number of people covered by health insurance or a public health system per 1000 population [47]. Aware that health insurance does not necessarily eliminate high out-of-pocket payments, human rights and civil society groups reacted with alarm to this substitution and called for urgent action to change the indicator for universal health coverage to one which provides a more meaningful measure of financial risk protection for the poorest groups [48]. A UN consultation on possible refinements of SDG indicators in November 2016 will consider replacing the current indicator with another proposed by WHO and the World Bank, “proportion of the population with large household expenditures (e.g. greater than 25%) on health as a share of total household expenditure or income” [49]. Oxfam and likely other human rights and civil society groups strongly support doing so [50].

WHO has recommended four policy initiatives to finance UHC: reducing out-of-pocket payments; maximizing mandatory prepayment; establishing large risk pools; and using general government revenues to cover those who cannot afford to contribute [51]. These approaches parallel recommendations made by Anand Grover when he was the Special Rapporteur for the right to health [52]. If adopted, the manner in which these policies are implemented will also have an impact on their effectiveness in making health care more accessible to poor and disadvantaged groups. For example, national policies to eliminate user fees in public health facilities without providing sufficient revenue to compensate facilities have been offset in a number of countries by the imposition of informal fees at the local level [53].

Moreover, not all prepayment mechanisms are pro-poor. Commercial health insurance is not. Social health insurance, a pooling mechanism funded by compulsory prepayments collected through individual and organizational contributions, which has been promoted by WHO, as for example in its 2010 report on health financing for UHC [54], as well as being favored by other public and private health funders, has worked to promote UHC in a number of high-income countries, but this model has had less success in low- and middle-income countries. It has the disadvantage of often excluding those who are not in the formal employment sector or who cannot afford the required health insurance payments. Importantly, no country has achieved anything close to UHC through relying on voluntary insurance contributions. Public financing has played a central role in all the UHC success stores to date [55].

#### Quality essential health care services

The UHC target in the SDGs identifies the need for access to quality essential health care services, but it does not provide guidance on what access entails. As the current Special Rapporteur for the right to health has noted, for universal health coverage to be consistent with the right to health it would have to meet the core requirements of availability, accessibility, and good quality. This would require that services be safely and geographically accessible without discrimination as well as being affordable. The health services would also need to be of sufficient quality, including in good working condition, and medically and scientifically appropriate for the population they cover [56].

Nor does the UHC target in the SDGs specify which health services should be provided for a country to be considered as having achieved UHC or which ones should have priority in the process of expanding coverage. Although General Comment 14 does not identify which health facilities, goods, and services should be provided in conjunction with achieving the right to health, it does reference the emphasis in the Committee’s General Comment No. 3 regarding the importance of providing essential primary health care [57]. It also notes that investments should not disproportionately favour expensive curative health services which are often accessible only to a small, privileged fraction of the population, rather than primary and preventive health care which can benefit a far larger proportion of the population [58]. In the absence of a similar caution in the SDGs, there is a risk that many countries may continue to invest scarce resources in the expensive tertiary care and high priced services which currently dominate the health budgets of many countries, to the exclusion of providing critical primary health services to a wider population. Doing so would be contrary to the three part strategy set forth by the WHO Consultative Group on Equity and Universal Health Coverage: First, categorize services into priority classes on the basis of such criteria as cost-effectiveness, priority to the worse off, and financial risk protection. Second, expand coverage for high-priority services to everyone. High-priority services are defined in the document as those that tend to be the most effective and to benefit the worse off; and third, as coverage is expanded, take special measures to ensure that disadvantaged groups, such as low-income groups and rural populations, are not left behind [59].

#### Financial cost sharing

Moving toward UHC will entail considerable financial costs. It should be noted that only eight of the 49 countries currently classified as being low-income are considered to have any prospect of generating sufficient funds to improve health coverage from domestic sources alone [60], and these countries, as well as many others, will require generous foreign aid to be able to progress toward UHC. A human rights approach recognizes the essential role of international cooperation and assistance in enabling resource poor states to implement economic, social and cultural rights and conceptualizes providing such aid as an obligation of more affluent countries. General Comment 14 reminds states, ‘For the avoidance of any doubt, the Committee wishes to emphasize that it is particularly incumbent on States parties and other actors in a position to assist, to provide ‘international assistance and cooperation, especially economic and technical’ which enable developing countries to fulfill their core and other obligations…’ [61].

A recent Chatham House report similarly recommends that to strengthen external financing for national health systems every country with sufficient capacity should contribute external financing. It envisions that net contributing countries would include all high-income countries and most upper middle-income countries. It proposes that high-income countries commit to provide external financing for health equivalent to at least 0.15% of GDP and that upper middle- income countries seek to progress toward the same contribution rate [62].

There is no such provision for external funding for the health targets in the SDGs. Although multilateral and bilateral aid was forthcoming to assist low-income countries progress toward the MDG goals, the prospects for low-income countries receiving needed generous financial aid to achieve the SDG goals is not good. The MDGs were framed as a compact between developed and developing countries with the affluent countries agreeing up front to provide financial aid to assist low-income countries. While rarely up to the level of pledges made by donor countries, international aid served as a key source of financing for implementing MDG health goals in many countries [63]. However, the SDGs were not conceptualized as a partnership, but as goals applicable to all countries, not just poor countries. Moreover, in contrast with the MDGs, which were forged in an atmosphere of global optimism in which prospects for increases in development assistance spending were bright, the SDGs were developed in a more pessimistic economic and political context. Many western countries are still suffering from the economic dislocations of the Great Recession with considerable economic insecurity, cuts in public services, and growing inequality [64]. Although the SDG Declaration acknowledges the important role international public finance plays in complementing the efforts of countries to mobilize domestic resources, especially in the poorest and most vulnerable countries with limited domestic sources, the SDG goals and targets do not offer any commitments of such aid. The Declaration just cites the pledge of some developed countries to achieve the target of 0.7% of gross national income for official development assistance [65], but this commitment, first made more than 30 years ago, has never been implemented. In contrast with the MDGs, the SDGs follow from the premise that each country has primary responsibility for its own economic and social development [66].

## Conclusion

UHC as framed in the SDGs falls short of human rights requirements. The most important of these is the failure to confer priority to providing access to health services to poor and disadvantaged communities in the process of expanding health coverage and in determining which health services to provide. Simply expanding health coverage, especially if doing so continues to exclude poor and vulnerable communities, is not sufficient from a human rights perspective. A related shortcoming is the failure of the proposed monitoring approach to incorporate disaggregation as proposed by WHO in order to produce statistics that would highlight inequalities in the implementation of the SDGs. Importantly, the approach to financial protection is inadequate to protect the poorest and most vulnerable groups from impoverishment from the cost of health services and the current indicator selected to monitor the status of the population along this line is inadequate. Unless those furthest behind are given priority, the SDGs, like the MDGs, are likely to leave the most disadvantaged and vulnerable communities behind.
